# The Balance in T Follicular Helper Cell Subsets Is Altered in Neuromyelitis Optica Spectrum Disorder Patients and Restored by Rituximab

**DOI:** 10.3389/fimmu.2019.02686

**Published:** 2019-11-19

**Authors:** Philippe Nicolas, Anne Ruiz, Alvaro Cobo-Calvo, Guillaume Fiard, Pascale Giraudon, Sandra Vukusic, Romain Marignier

**Affiliations:** ^1^Service de Neurologie, Sclérose en Plaques, Pathologies de la Myéline et Neuro-Inflammation, et Centre de Référence des Maladies Inflammatoires Rares du Cerveau et de la Moelle (MIRCEM), Hôpital Neurologique Pierre Wertheimer, Hospices Civils de Lyon, Bron, France; ^2^Centre de Recherche en Neurosciences de Lyon, INSERM U1028-CNRS UMR5292-UCBL, Bron, France; ^3^NeuroBioTec, Hôpital Neurologique Pierre Wertheimer, Bron, France

**Keywords:** neuromyelitis optica spectrum disorder, T follicular helper cells, T follicular regulatory cells, B cells, rituximab

## Abstract

Neuromyelitis optica spectrum disorder (NMOSD) is a rare and severe auto-immune disease of the central nervous system driven by pathogenic antibodies mainly directed against aquaporin-4 (AQP4-Ab). Treatment of NMOSD currently relies on immunosuppressants (mycophenolate mofetil, azathioprine) or B-cell-depleting therapy (rituximab). B-cell differentiation into antibody-producing cells requires T follicular helper cells (Tfh). There are several Tfh subsets that differentially affect B-cell differentiation; Tfh2 and Tfh17 subsets strongly support B-cell differentiation. By contrast, Tfh1 lack this capacity and T follicular regulatory cells (Tfr), inhibit B-cell differentiation into antibody-producing cells. We performed a broad characterization of circulating Tfh subsets in 25 NMOSD patients and analyzed the impact of different treatments on these subsets. Untreated NMOSD patients presented a Tfh polarization toward excessive B-helper Tfh subsets with an increase of Tfh17 and (Tfh2+Tfh17)/Tfh1 ratio and a decrease of Tfr and Tfh1. Rituximab restored the Tfh polarization to that of healthy controls. There was a trend toward a similar result for azathioprine and mycophenolate mofetil. Our results suggest that NMOSD patients present an impaired balance in Tfh subsets favoring B-cell differentiation which may explain the sustained antibody production. These findings provide new insights into the pathophysiology of NMOSD, and further suggest that Tfh and Tfr subsets could be considered as potential therapeutic target in NMOSD because of their upstream role in antibody production.

## Introduction

Neuromyelitis optica spectrum disorder (NMOSD) is a rare auto-immune disease of the central nervous system (CNS) characterized by recurrent attacks of optic neuritis and transverse myelitis leading to potential severe disability (1). Treatment of NMOSD currently relies on immunosuppressants (mycophenolate mofetil—MFM, azathioprine—AZA) or B-cell-depleting therapy (rituximab—RTX) (2, 3).

Although the pathophysiology of NMOSD is not fully understood, 80% of patients have circulating pathogenic auto-antibodies targeting aquaporin-4 (AQP4-Ab) that is expressed by astrocytes (4–7). More recently, auto-antibodies targeting the myelin oligodendrocyte glycoprotein (MOG) have been identified in a subset of AQP4-IgG negative NMOSD patients (8–11).

These antibodies are produced by plasmablasts and long-lived plasma cells that derive from B-cells with the support of T follicular helper cells (Tfh) (12). Peripheral human Tfh are characterized by a CXCR5+ CD45RA– CD4+ phenotype, and comprise several subsets that differentially affect B-cell differentiation into antibody producing cells (13). Tfh1 (CXCR3+ CCR6–) lack capacity to help naïve B-cells to produce antibodies (13), Tfh2 (CXCR3– CCR6) and Tfh17 (CXCR3– CCR6+) are, on the contrary, strong B-helper Tfh subsets (14); the (Tfh2+17)/Tfh1 ratio has been proposed to characterize the “helper capacities” of Tfh (15). T follicular regulatory cells (Tfr; Foxp3+, or CD25^high^ CD127^low^) inhibit the differentiation of B-cell into antibody-producing cells (16).

Several autoimmune diseases are associated with either an increase in strong B-helper Tfh subsets (Tfh2 and Tfh17) and/or a decrease in non B-helper Tfh subsets (Tfh1) or regulatory subset (Tfr) (15, 17–19). Only a few studies have focused on circulating Tfh in NMOSD (20–23). Circulating Tfh were found to be increased in NMOSD patients, and even more during relapses (20–22). Tfh cell frequency decreased under methylprednisolone (20) and rituximab treatments (22). However, a large and prospective analysis of Tfh subsets (Tfh1, Tfh2, Tfh17, Tfr) in NMOSD is lacking.

In the present study, we performed a broad characterization of circulating Tfh subsets (Tfh1, Tfh2, Tfh17, Tfr) in a prospective cohort of NMOSD patients. We also evaluated the effects of different treatments on Tfh subpopulations.

## Materials and Methods

### NMOSD Patients and Healthy Controls

Samples were obtained from 25 NMOSD patients, consecutively admitted at the Hôpital Neurologique (Lyon, France), from January to August 2017. Inclusion criteria were patients aged over 16 years old and fulfilling the 2015 IPND diagnostic criteria for NMOSD (7), whatever the serostatus, disease course and disease modifying drug. Exclusion criteria were (i) previous corticosteroid treatment within 3 months before sampling and (ii) another active immune disorder, infection or cancer. The patients who had received another disease modifying drug within the previous year were excluded from the treatment analysis. Clinical data was noted during routine clinical visits using standardized case report forms and registered in the French NOMADMUS database. Age, sex, disease duration, number and type of relapse were recorded. A relapse was defined as a new neurological symptom lasting at least 24 h and accompanied by new neurological examination finding and new lesions on MRI. The clinical remission was defined as both neurological symptoms and neurological examination signs that remained stable for at least 30 days from last relapse. Serostatus for AQP4-Ab and MOG-Ab was assessed by cell-based assay in the lyon neuroscience research center (Lyon, France), as previously reported (24, 25). The degree of disability was assessed by the Expanded Disability Status Scale (EDSS). Current and former treatments were recorded. None of the 25 NMOSD patients received methylprednisolone within 3 months before sampling. Two patients were excluded from the treatment analysis because they had received another disease modifying drug within the previous year (Supplementary Table 1).

Samples from 12 healthy controls (HC) were collected in parallel at the Hôpital Neurologique (Lyon, France), from January to August 2017. None of them had a history of disease or infection within the previous 3 months and none received any treatment within the previous 3 months; all were immunosuppressant drug naive.

### Human Samples and PBMC Isolation

Heparinized blood from NMOSD patients and HC were collected from January to August 2017 and analyzed within 24 h.

Peripheral blood mononuclear cells (PBMC) were isolated by diluting blood (1:1) with phosphate-buffered saline (PBS) 1X and layered over Ficoll-Paque Premium 1084® (Sigma-Aldrich Corporation, St. Louis, MO, USA) according to the manufacturer's instruction.

### Flow Cytometry

PBMC were labeled in staining buffer (0.05% azide, 2.5% FCS, 2 mM EDTA, PBS1X) with anti-human antibody (anti-CD3, anti-CD4, anti-CD45RA, anti-CXCR5, anti-CD25, anti-CD127, anti-CCR6, and anti-CXCR3). This panel was design based on fluorochrome brightness and antigen density and allowed the analysis of total Tfh, Tfh1, Tfh2, Tfh17, and Tfr (Reference for antibodies are presented in Supplementary Table 4). Cells were incubated 30 min at 4°C and washed 2 times with PBS 1X. Cell viability was determined upstream using trypan blue and more than 90% of cells were viable.

**Figure 1:**
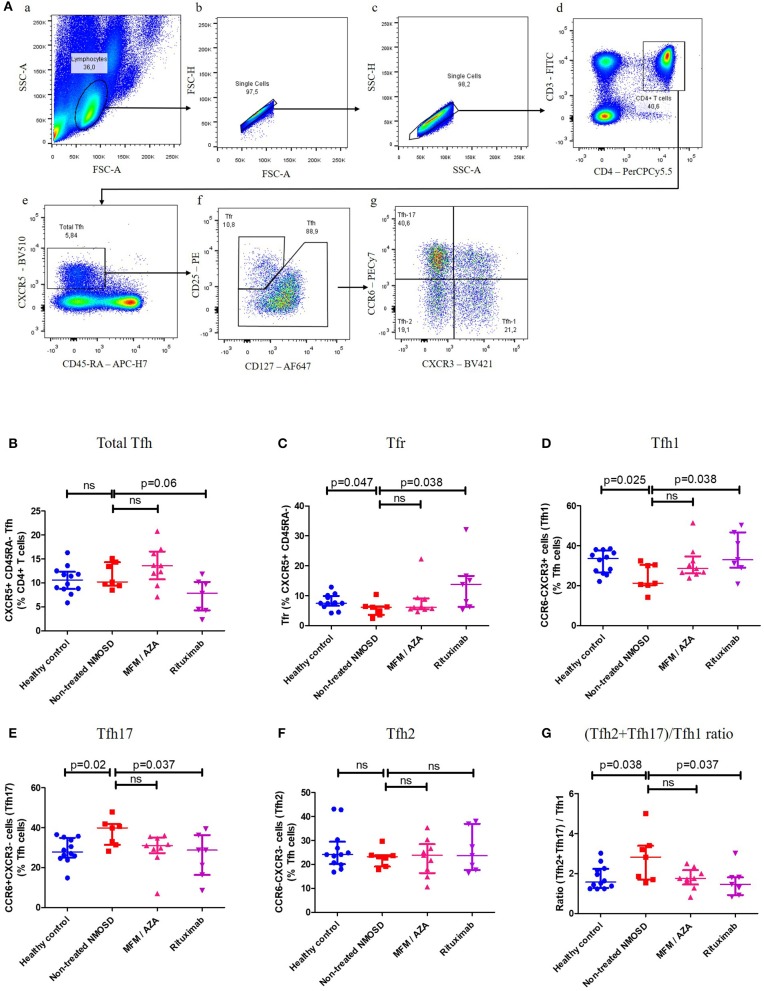
Tfh subsets distribution in healthy controls, non-treated NMOSD patients and treated NMOSD patients. **(A)** Gating strategy for Total Tfh, Tfr, Tfh1, Tfh2, and Tfh17 subsets. (a) Sideward scatter-A (SSC-A) and Forward scatter-A (FSC-A) were used for lymphocytes gating. (b,c) FSC-H and FSC-A as well as SSC-H and SSC-A were used to gate singlets. (d) CD4+ T cells were CD3+CD4+. (e) Total Tfh were CXCR5+CD45RA- within CD4 T cells. (f) T follicular Regulatory cells (Tfr) were CD25^high^CD127^low^and (non-regulatory) Tfh were CD25^negative to low^ CD127^low to high^ (g) Among those Tfh cells, CXCR3 and CCR6 chemokine receptors were used to differentiate: Tfh1 (CXCR3+CCR6–), Tfh2 (CXCR3–CCR6–), and Tfh17 (CXCR3–CCR6+). **(B)** Proportion of total Tfh (CXCR5+ CD45RA–) within CD4+ T cells in healthy controls (HC) (bleu dots), non-treated NMOSD patients (red squares), MFM/AZA-treated NMOSD patients (pink triangle), and RTX-treated NMOSD patients (purple triangle). Kruskal–Wallis test: *p* = 0.027. **(C)** Proportion of Tfr within CXCR5+ CD45RA– CD4+ T cells in HC, non-treated NMOSD patients, MFM/AZA-treated NMOSD patients and RTX-treated NMOSD patients. Kruskal-Wallis test: *p* = 0.074. **(D)** Proportion of Tfh1 within Tfh cells in HC, non-treated NMOSD patients, MFM/AZA-treated NMOSD patients and RTX-treated NMOSD patients. Kruskal–Wallis test: *p* = 0.074. **(E)** Proportion of Tfh17 within Tfh cells in HC, non-treated NMOSD patients, MFM/AZA-treated NMOSD patients and RTX-treated NMOSD patients. Kruskal–Wallis test: *p* = 0.069. **(F)** Proportion of Tfh2 within Tfh cells in HC, non-treated NMOSD patients, MFM/AZA-treated NMOSD patients and RTX-treated NMOSD patients. Kruskal–Wallis test: *p* = 0.8 **(G)** (Tfh2+Tfh17)/Tfh1 ratio in HC, non-treated NMOSD patients, MFM/AZA-treated NMOSD patients and RTX-treated NMOSD patients. Kruskal–Wallis test: *p* = 0.11. Each data point represents an individual subject. Horizontal lines show median ± IQR.

Cell suspensions were analyzed on FACS Canto II (Beckton-Dickinson, San Diego, CA, USA) equipped with 3 lasers (488, 633, 405 nm) using the BD FACS-Diva^TM^ software. FACS analyses were done blinded and compensation were computed by the FlowJo software (version 10 by Flowjo LLC, OR, USA). Compensation matrix is presented in Supplementary Table 5. The same gating strategy has been used for all data files (Figure 1A). To determinate gates Fluorescence Minus One (FMO), control and back-gating has been used.

Variability was limited by analyzing samples on the same cytometer and by strict adherence to sample handling and staining protocol. At each analysis, stained and unstained cells were included. To monitor cytometer performance, instrument setup and performance tracking was done on a daily basis, using BD cytometer setup and tracking beads. Application settings were created on Diva for the same panel.

### Standard Protocol Approvals, Registrations, and Patient Consents

The study was conducted in accordance with the French law relative to clinical non-interventional research. According to the French law on Bioethics (July 29, 1994; August 6, 2004; and July 7, 2011, Public Health Code), the patients' written informed consent was collected. Moreover, data confidentiality was ensured in accordance with the recommendations of the French commission for data protection (*Commission Nationale Informatique et Liberté*, CNIL decision DR-2014-558).

### Statistical Analysis

In the present study, we reproduced previous results suggesting that RTX inhibits Tfh increase in NMOSD as well as the impact of RTX on Tfh frequency (22) by a confirmatory analysis. We further performed an exploratory analysis after considering consistent and coherent hypothesis pre-tests based on the similarly altered distribution of Tfh subsets in other antibody-mediated autoimmune disorders such as myastenia gravis, guillain-barré syndrome or lupus (15, 17–19). In these diseases, Tfh subsets distribution is altered toward an increase of Tfh2 and/or Tfh17 and a decrease of Tfh1 and Tfr. We hypothesized that in NMOSD, Tfh subsets distribution would be similar. Thus, such hypothesis pre-test will increase the probability pre-test to find a real positive result, in other words, a low type 1 error.

Continuous variables were reported as median and interquartile ranges (IQR). First, we performed a Kruskal Wallis non-parametric test to evaluate differences among more than two groups and we used *U*-Mann Whitney's non-parametric test if appropriate, to compare group by group. We first compared Tfh subsets distribution between non-treated NMOSD patients and HC. Then we compared Tfh subsets distribution between non-treated NMOSD, AZA/MFM treated NMOSD and RTX-treated NMOSD. Same analyses were performed regarding serostatus (MOG, AQP4 and double seronegative) and EDSS at sampling categorized in EDSS ≤2.5 and ≥3.0. Statistical significance was set at two-tailed *p* < 0.05, and analyses conducted using GraphPad Prism Software (Version 5.0b for Windows, GraphPad Software, San Diego, CA, 31 USA).

## Results

Demographic, clinical, and biological characteristics of NMOSD patients and HC are detailed in Table 1 and Supplementary Tables 1–3.

**Table 1:** Demographic characteristics of NMOSD patients and HC.

**Characteristics**	**NMOSD**	**HC**
*n*	25	12
Age (range), years	35 (16-81)	27 (25-31)
Female, *n* (%)	19 (76)	6 (50)
ARR	0.38	–
EDSS	2	–
Median disease duration in months (Range)	72 (3-240)	–
AQP4-IgG, *n* (%)	13 (52)	–
MOG-IgG, *n* (%)	9 (36)	–
Double seronegative, *n* (%)	3 (12)	12 (100)
Relapsing, *n* (%)	2 (8)	–
Remitting, *n* (%)	23 (92)	
No treatment, *n* (%)	8 (32)	12 (100)
Mycophenolate mofetil or azathioprine, *n* (%)	10 (40)	–
Rituximab, *n* (%)	7 (28)	–

*Data are expressed as median, unless otherwise stated. NMOSD, neuromyelitis optica spectrum disorder; HC, healthy controls; AAR, annualized relapse rate; EDSS, expanded disability status scale; AQP4-IgG, Anti-AQP4 antibody positive patients; MOG-IgG, anti-MOG antibody positive patients*.

### Tfh Subsets Distribution Is Altered in Non-treated NMOSD Patients

When analyzed all together, NMOSD patients presented no significant difference compared to HC in terms of total Tfh, Tfr, Tfh1, Tfh2, Tfh17 proportions and (Tfh2+Tfh17)/Tfh1 ratio (Supplementary Figure 1). We did not find any difference in Tfh subset distribution according to the serostatus and the EDSS score (Supplementary Figures 2, 3). Two patients were relapsing, but the low number doesn't allow statistical analysis.

NMOSD patients were then clustered into three different groups based on their treatments; i.e., non-treated, MFM/AZA and RTX-treated NMOSD patients. There was no significant difference in the proportion of total Tfh cells (CXCR5+CD45RA–) within CD4+ T cells, between non-treated NMOSD patients and HC [Med (IQR): 10.2% (9.4–14.3) vs. 10.6% (8.7–12.3); *p* = 0.47, Figure 1B]. Non-treated NMOSD patients had a significantly lower proportion of Tfr compared to HC [Med (IQR): 6.1% (3.6–6.3) vs. 7.5% (6.6–9.9); *p* = 0.047, Figure 1C]. Compared to HC, non-treated NMOSD patients had a significantly lower proportion of Tfh1 [Med (IQR): 21.2% (20.2–30.4) vs. 33.6% (26.5–37.6); *p* = 0.025, Figure 1D] a significantly higher proportion of Tfh17 [Med (IQR): 39.9% (31.4–41.9) vs. 27.9% (24.9–34.9); *p* = 0.02, Figure 1E] and no significant difference in the proportion of Tfh2 [Med (IQR): 23.2% (19.1–23.8) vs. 24.10% (20.1–29.4); *p* = 0.3, Figure 1F]. The (Tfh2+Tfh17)/Tfh1 ratio was significantly higher in non-treated NMOSD patients compared to HC [Med (IQR): 2.8 (1.7–3.4) vs. 1.6 (1.3–2.2); *p* = 0.038, Figure 1G].

### Rituximab Restored Tfh Subsets Distribution in NMOSD Patients

Compared to non-treated patients, in RTX-treated patients there was a trend toward a lower proportion of total Tfh [Med (IQR): 7.8% (4.3–1.2) vs. 10.2% (9.4–14.3); *p* = 0.47, Figure 1B], a significantly higher proportion of Tfr [13.8% (6.3–16.6) vs. 6.1% (3.6–6.3); *p* = 0.038, Figure 1C] and Tfh1 [33% (29–46.7) vs. 21.2% (20.2–30.4); *p* = 0.025, Figure 1D]; there was a lower proportion of Tfh17 [28.7% (16.4–36.3) vs. 39.9% (31.4–41.9); *p* = 0.038, Figure 1E] and no significant difference in the proportion of Tfh2 [23.7% (17.7–36.9) vs. 23.2% (19.1–23.8); *p* = 0.8, Figure 1F]. The (Tfh2+Tfh17)/Tfh1 ratio was significantly lower in RTX-treated patients compared to non-treated NMOSD patients [1.5 (0.9–1.8) vs. 2.8 (1.7–3.4); *p* = 0.038, Figure 1G].

Compared to non-treated patients, in patients treated by AZA or MFM there was a trend toward a lower proportion of Tfh17, (Tfh2+Tfh17)/Tfh1 ratio, and a higher proportion of Tfh1 and Tfr (all non-significant; Figure 1).

## Discussion

In a prospective cohort of NMOSD patients, we found that Tfh subsets were unbalanced toward an excessive B-helper Tfh phenotype [Tfh17 and (Tfh2+Tfh17)/Tfh1 ratio]. Interestingly, RTX restore this imbalance.

We confirm herein that Tfh population is altered in non-treated NMOSD patients (20–22) but only at the subset level with a higher proportion of Tfh17, a lower proportion of the Tfh1, a higher (Tfh2+Tfh17)/Tfh1 ratio, and a lower proportion of Tfr, compared with HC. These findings suggest that the altered balance in Tfh subsets may facilitate B cell differentiation into antibody producing cells in NMOSD patients. The data are in accordance to recent studies on other autoimmune diseases (13, 15, 17, 18, 26, 27). However, we did not explore the correlation between the alteration of Tfh subsets distribution and the frequency of plasmablasts or AQP4-Ab and MOG-Ab titers and such analyses should be performed in future studies.

The causes of the impaired balance in Tfh subsets in NMOSD remains elusive. The differentiation of naïve T-cells into different Tfh subsets relies on specific signals provided by antigen presenting cells (28). NMOSD patients might present an abnormal antigen presentation, leading to an impaired balance in Tfh subsets. This abnormal presentation could be determined by genetics [MHC polymorphism (29), polymorphism in innate receptors, differences in cytokine production (28)] and/or by the environment [previous infection (30)]. One could argue that the altered balance in Tfh subsets observed in the periphery is the visible consequence of a preferential recruitment of Tfh1 cells in the central nervous system. However, there is no data regarding the presence of Tfh1 in the parenchyma or CSF in NMOSD patients.

RTX effectiveness in NMOSD has been related to the decrease of antibody production. However, there is no clear evidence of such decrease (31, 32) and CD20, the target of RTX, is not expressed by antibody-producing cells. The present study suggests an alternative mechanism of action; RTX could restore the altered balance in Tfh subsets in NMOSD patients. Zhao et al. have already shown that the frequency of circulating Tfh cells (CD3+CD4+CXCR5+PD-1+) was significantly decreased under RTX in NMOSD (22). They also showed *ex vivo*, that Tfh maintenance was dependent on B cell, throw IL6 signaling and direct contact (22) and suggested a positive feedback loop between B cells and Tfh cells, that could be disrupted by RTX. Our data are in keeping with these results and further suggest that this loop could be even more sharply modulated. One could hypothesize that activated B-cells may be responsible for the maintenance of strong B-helper Tfh populations (Tfh2, Tfh17) and the downregulation of both the non-helper population (Tfh1) and regulatory population (Tfr). B-cell depletion under RTX would release this selection pressure of Tfh subsets favoring B-cell activation. One cannot, however, exclude a more direct effect of RTX on Tfh cells; recent evidence strongly supports a role of RTX beyond B-cell depletion (33). The importance of CD20+ T-cells is still a matter of debate, but 3–5% of circulating T-cells express a low level of CD20 and have been shown to be depleted by RTX (34). Further studies are needed to assess the relevance of CD20 expression on Tfh subsets in RTX-treated NMOSD patients.

Long-term exposure to RTX can lead to severe side-effects, including infection related to hypogammaglobulinemia (35). In addition, some patients persist in relapsing despite B-cell depletion characterized by lack of CD19+CD27+ cells (36). Biomarkers to identify such patients for whom RTX is inefficient or for monitoring the timing re-infusion are lacking. Since RTX is able to alter Tfh1, Tfh17, and Tfr subsets, these could be potential biomarkers to discriminate responder and non-responder patients. Longitudinal studies with larger sample sizes are needed to evaluate the relevance of such biomarkers.

Inclusion of non-AQP4-IgG+ NMOSD patients is arguable. Indeed, AQP4-IgG+ and MOG-IgG+ NMOSD patients display some pathophysiological specificities as they are characterized by an astrocytopathy and an oligodendrogliopathy, respectively. However, even if their targets are different, both entities are considered as antibody-mediated CNS disorders, sharing a similar phenotype. In the present exploratory study, all the patients met the 2015 IPND diagnostic criteria for NMOSD. Indeed, until the criteria of NMOSD are not modified, patients that are seronegative or with MOG-IgG and meet the current criteria must be diagnosed with NMOSD. Further studies including more patients with homogeneous treatments are required to determine if Tfh subsets are differentially affected according to NMOSD serostatus.

The present study has some limitations. There was a trend toward an impaired balance in MFM or AZA-treated patients similar to RTX-treated patients. We assume that these results did not reach significance because of the lack of statistical power. Thus, further studies are needed to assess whether or not AZA and MFM affect Tfh subset distribution in NMOSD patients. HC were not age or gender-matched, resulting in some demographical differences between NMOSD patients and HC. However, we assume that this did not affect the conclusions of the study since differences regarding the mean of age were not relevant and there was no significant difference in the proportion of Tfh between male and female.

Finally, our work focuses on Tfh subsets (Tfh1, Tfh2, Tfh17, and Tfr) and did not take into account Tfh activation status nor Tfh memory phenotype that also affect B cell differentiation. On the one hand, Tfh activation status is mainly characterized by PD1 and ICOS expression (37). Both PD1+ and ICOS+ Tfh are increased in NMOSD and especially during relapses (20–22), but the exploration of PD1 and ICOS expression on Tfh subsets are lacking in NMOSD. On the other hand, the memory phenotype of Tfh is mainly determined by CCR7 expression (37). Effector memory Tfh (CCR7^low^) are considered as active Tfh and are increased in NMOSD patients and correlate with the plasma level of AQP4-Ab and the annual relapse rate (21). On the contrary, central memory Tfh (CCR7^high^) are considered as quiescent but have the ability to migrate to germinal centers (38) as well as in the CNS (39) where they could be involved in intrathecal antibody production in NMOSD (21). CCR7 expression on Tfh subsets have never been explored in NMOSD. In the light of our findings, exploring the expression of at least those 3 activation/memory markers (PD1, ICOS, CCR7) on the four Tfh subsets (Tfh1, Tfh2, Tfh17, and Tfr) is crucial in order to decipher precisely the role of Tfh in NMOSD.

## Conclusion

The present study confirms the alteration of Tfh in NMOSD. We show that the balance in Tfh subsets is altered toward an excessive B-helper phenotype which could be responsible for the chronic B-cell activation and antibody production. This impaired balance can be reversed by rituximab. Tfh and Tfr subsets, because of their upstream position in the antibody production, could therefore be considered as prime candidates for future therapeutic strategies in NMOSD. Our work opens the way to a large-scale longitudinal study, monitoring Tfh subsets distribution, including activation and memory markers, in the blood and in the CSF of NMOSD patients with homogeneous serostatus and treatments.

## Data Availability Statement

All datasets generated for this study are included in the article/Supplementary Material.

## Ethics Statement

The study was conducted in accordance with the French law relative to clinical non interventional research. According to the French law on Bioethics (July 29, 1994; August 6, 2004; and July 7, 2011, Public Health Code), the patients' written informed consent was collected. Moreover, data confidentiality was ensured in accordance with the recommendations of the French commission for data protection (Commission Nationale Informatique et Liberté, CNIL decision DR-2014-558).

## Author Contributions

RM, AR, and PG: study conception and design. PN, AR, AC-C, PG, SV, and RM: literature search. PN, AR, GF, SV, and RM: acquisition of data. PN, AR, AC-C, GF, PG, SV, and RM: data analysis and interpretation. PN, AC-C, and RM: figures. PN, AR, AC-C, and RM: drafting the article. PN, AR, AC-C, GF, PG, SV, and RM: critical revision of the article and final approval of the version to be published.

### Conflict of Interest

The authors declare that the research was conducted in the absence of any commercial or financial relationships that could be construed as a potential conflict of interest.

## Abbreviations

AQP4: aquaporin-4
AZA: azathioprine
CNS: central nervous system
EDSS: expanded disability status scale
IPND: International panel for NMO diagnosis
MFM: mycophenolate mofetil
MOG: myelin oligodendrocyte glycoprotein
NMOSD: neuromyelitis optica spectrum disorder
RTX: rituximab
Tfh: T follicular helper cells
Tfr: T follicular regulatory cells.
