# Supplementary figures and images for: The Balance in T Follicular Helper Cell Subsets Is Altered in Neuromyelitis Optica Spectrum Disorder Patients and Restored by Rituximab
Source: Front Immunol. 2019 Nov 19;10:2686. doi: 10.3389/fimmu.2019.02686 (PMC6877601; doi:10.3389/fimmu.2019.02686)

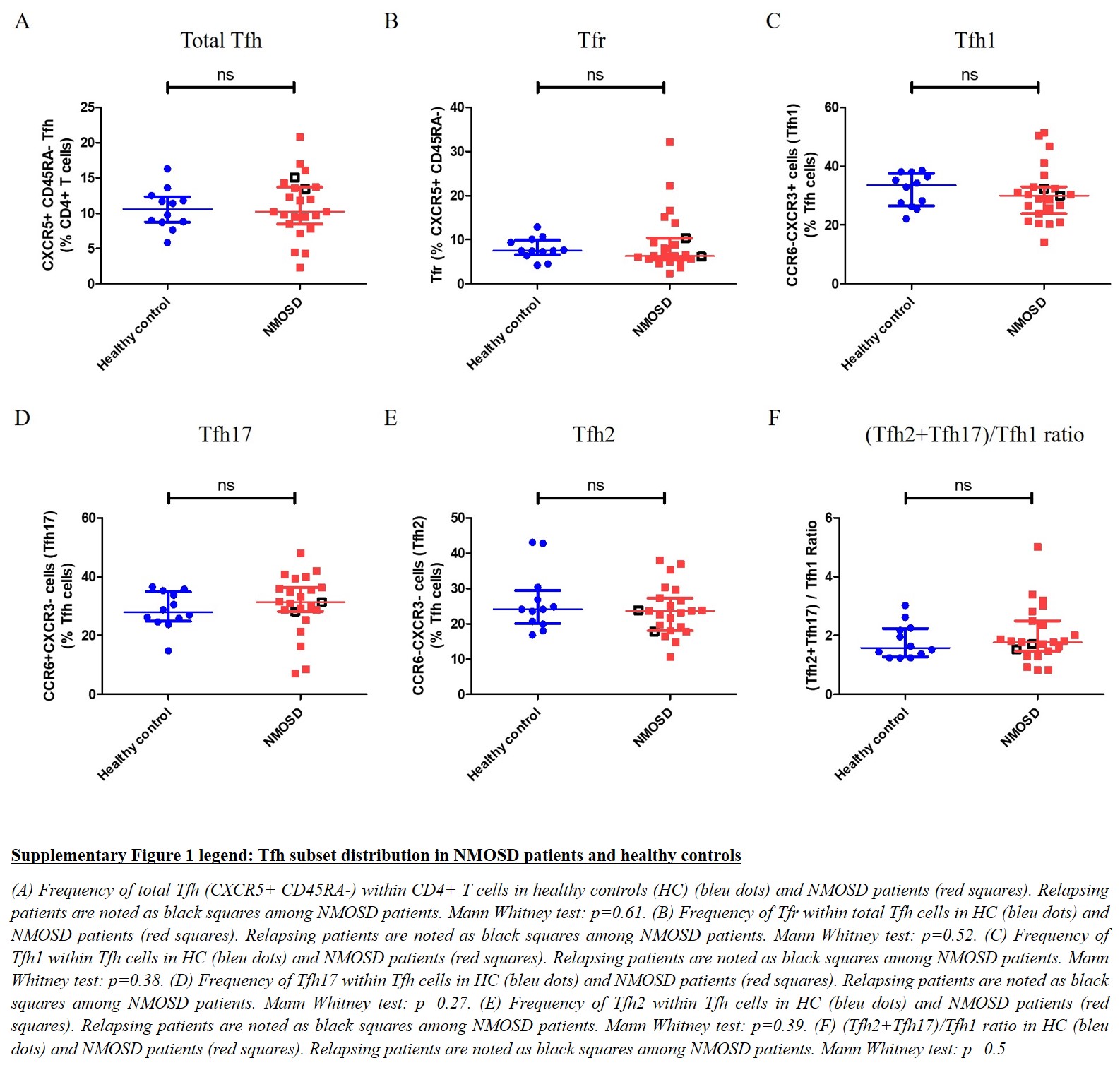

Supplement: Supplementary file 1 [file Image_1.jpg]

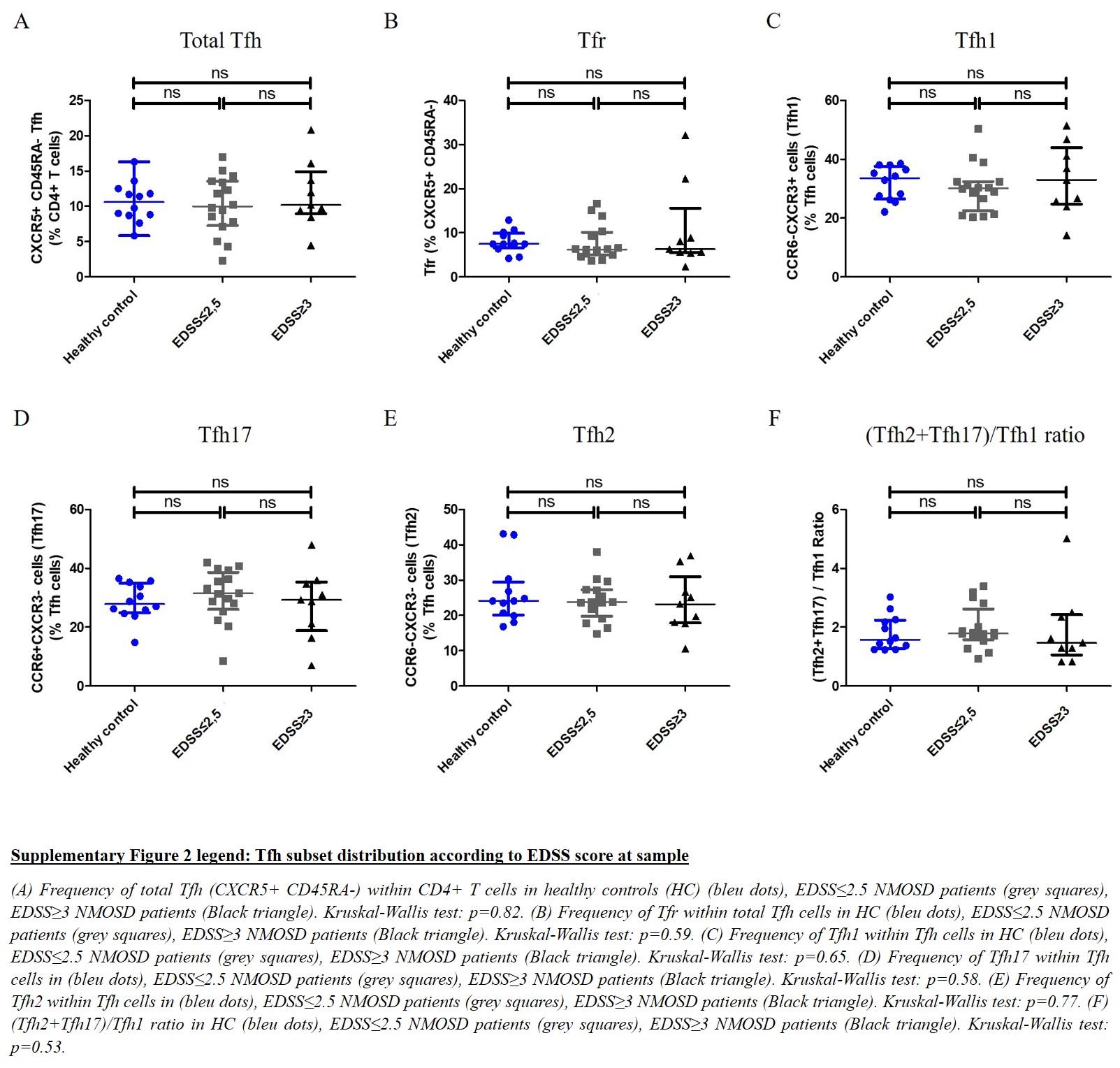

Supplement: Supplementary file 2 [file Image_2.jpg]

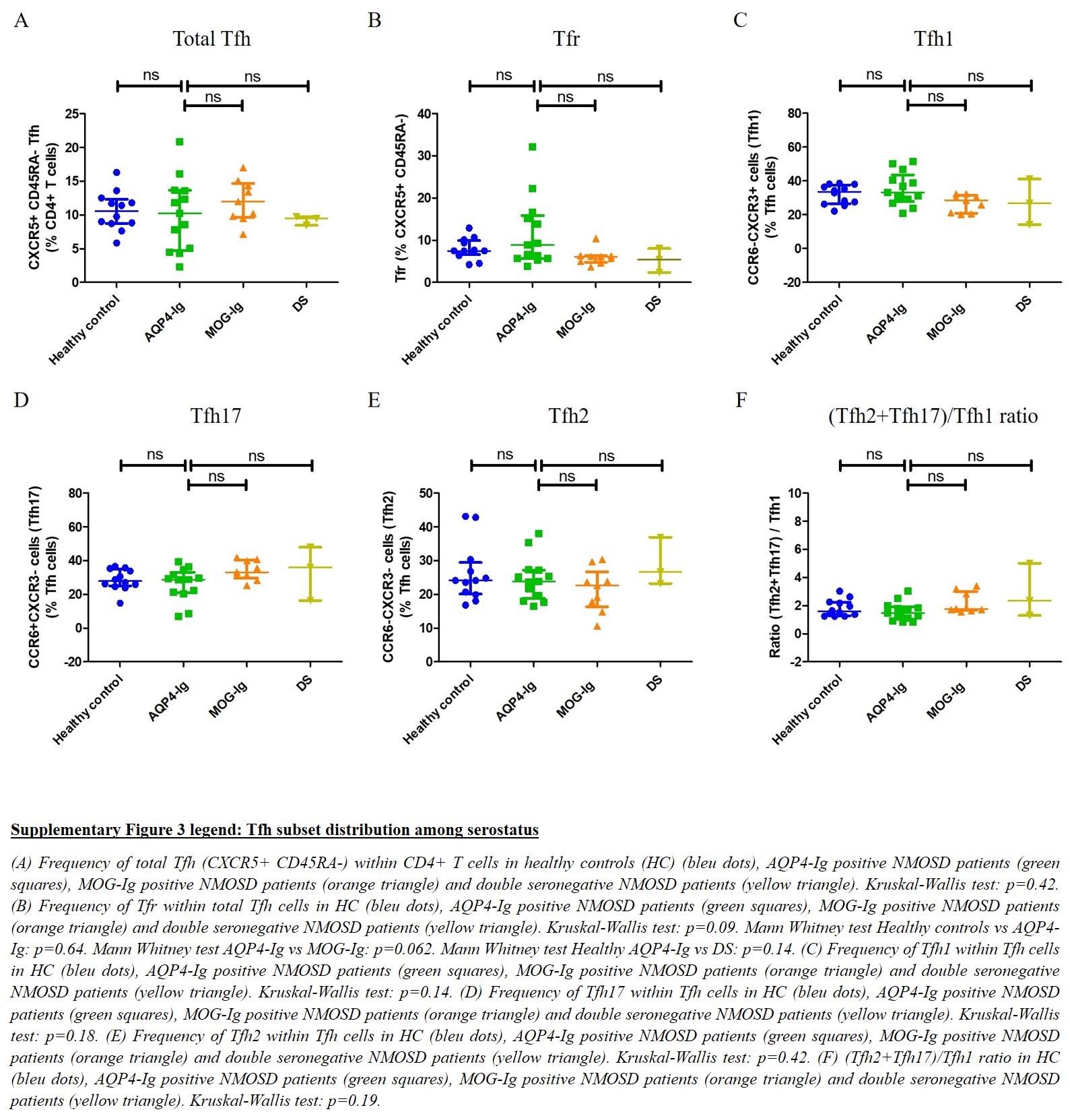

Supplement: Supplementary file 3 [file Image_3.jpg]
