# Supplementary material for: The Balance in T Follicular Helper Cell Subsets Is Altered in Neuromyelitis Optica Spectrum Disorder Patients and Restored by Rituximab
Source: Front Immunol. 2019 Nov 19;10:2686. doi: 10.3389/fimmu.2019.02686 (PMC6877601; doi:10.3389/fimmu.2019.02686)
Supplement: Supplementary file 4 [file Table_1.docx]

Supplementary table 1. Detailed patients’ clinical characteristics

| Patient | Age | Gender | Disease duration (months) | Total number of relapses | ON | TM | Ongoing Treatment | EDSS at sample | Relapse | Serostatus |
| --- | --- | --- | --- | --- | --- | --- | --- | --- | --- | --- |
| 1 | 16 | M | 12 | 3 | 2 | 0 | NT | 0 | No | MOG+ |
| 2 | 24 | F | 72 | 3 | 1 | 2 | RTX | 3 | No | AQP4+ |
| 3 | 36 | M | 216 | 2 | 1 | 1 | MFM | 6 | No | DN |
| 4 | 34 | F | 108 | 3 | 0 | 3 | AZA | 0 | No | AQP4+ |
| 5 | 33 | F | 132 | 3 | 1 | 1 | NT | 0 | No | MOG+ |
| 6 | 35 | M | 29 | 3 | 3 | 0 | NT | 2 | Yes | MOG+ |
| 7 | 49 | F | 66 | 1 | 0 | 1 | MFM | 1 | No | AQP4+ |
| 8 | 55 | F | 100 | 2 | 0 | 2 | MFM | 4 | No | AQP4+ |
| 9 | 24 | F | 180 | 2 | 1 | 1 | RTX | 2 | No | AQP4+ |
| 10 | 42 | F | 204 | 5 | 4 | 1 | RTX | 1 | No | AQP4+ |
| 11 | 38 | F | 23 | 2 | 2 | 0 | NT | 0 | No | MOG+ |
| 12 | 46 | M | 14 | 1 | 0 | 1 | NT | 0 | No | MOG+ |
| 13 | 30 | F | 29 | 1 | 0 | 1 | AZA | 0 | No | MOG+ |
| 14 | 23 | F | 39 | 2 | 2 | 0 | AZA | 2 | No | MOG+ |
| 15 | 20 | F | 65 | 10 | 2 | 5 | NT | 2 | No | AQP4+ |
| 16 | 76 | F | 172 | 4 | 0 | 4 | MFM | 6,5 | No | AQP4+ |
| 17 | 81 | F | 21 | 5 | 2 | 3 | RTX | 6 | No | AQP4+ |
| 18 | 53 | F | 28 | 1 | 0 | 1 | RTX | 2 | No | AQP4+ |
| 19 | 31 | M | 193 | 5 | 1 | 0 | RTX | 4 | No | DN |
| 20 | 31 | F | 36 | 1 | 0 | 1 | MFM | 2 | No | AQP4+ |
| 21 | 63 | F | 240 | 4 | 1 | 3 | MFM | 6 | No | AQP4+ |
| 22 | 51 | M | 96 | 2 | 1 | 1 | NT | 4 | No | DN |
| 23 | 32 | F | 3 | 2 | 1 | 1 | NT | 0 | Yes | MOG+ |
| 24 | 60 | F | 96 | 3 | 1 | 2 | MFM | 3 | No | MOG+ |
| 25 | 30 | F | 97 | 3 | 2 | 1 | RTX | 1 | No | AQP4+ |

*ON: Optic neuritis; TM: Transverse myelitis; EDSS:* *Expanded Disability Status Scale; M: male; F: Female; NT: non-treated; RTX: Rituximab; MFM: Mycophenolate mofetil; AZA: Azathioprine. Patient n°4 and n°15 were excluded from the treatment analysis because they had received rituximab during the previous year; DN: Double seronegative patients*
