# Supplementary material for: The Balance in T Follicular Helper Cell Subsets Is Altered in Neuromyelitis Optica Spectrum Disorder Patients and Restored by Rituximab
Source: Front Immunol. 2019 Nov 19;10:2686. doi: 10.3389/fimmu.2019.02686 (PMC6877601; doi:10.3389/fimmu.2019.02686)
Supplement: Supplementary file 5 [file Table_2.docx]

| Patient | Tfh | Tfh1 | Tfh2 | Tfh17 | Tfr | Ratio |
| --- | --- | --- | --- | --- | --- | --- |
| 1 | 14,3 | 20,5 | 29,6 | 39,9 | 3,61 | 3,39 |
| 2 | 10,2 | 33 | 19,7 | 28,8 | 6,27 | 1,47 |
| 3 | 9,44 | 26,7 | 26,6 | 36 | 5,4 | 2,34 |
| 4 | 5,03 | 40,5 | 25,4 | 20,3 | 5,66 | 1,13 |
| 5 | 10,2 | 30,4 | 23,6 | 33,1 | 6,36 | 1,87 |
| 6 | 13,4 | 30 | 17,8 | 28,2 | 10,4 | 1,53 |
| 7 | 12,3 | 30,3 | 23,8 | 29,7 | 6,55 | 1,77 |
| 8 | 13,7 | 51,4 | 35,3 | 7,01 | 8,9 | 0,82 |
| 9 | 2,33 | 31,1 | 27,3 | 28,7 | 15,1 | 1,80 |
| 10 | 7,81 | 29 | 16,4 | 36,3 | 16,6 | 1,82 |
| 11 | 9,45 | 21,2 | 19,1 | 40,7 | 4,97 | 2,82 |
| 12 | 9,78 | 20,2 | 22,6 | 41,9 | 6,28 | 3,19 |
| 13 | 17 | 28,5 | 14,8 | 35,5 | 4,62 | 1,76 |
| 14 | 7,11 | 32,4 | 30,3 | 25,3 | 6,1 | 1,72 |
| 15 | 8,54 | 38,9 | 27,1 | 22,3 | 3,77 | 1,27 |
| 16 | 20,8 | 23,9 | 25,1 | 34,7 | 5,67 | 2,50 |
| 17 | 4,48 | 46,7 | 17,7 | 21,3 | 32,1 | 0,84 |
| 18 | 4,29 | 20,9 | 23,7 | 39,4 | 13,8 | 3,02 |
| 19 | 9,73 | 41,1 | 36,9 | 16,4 | 8,06 | 1,30 |
| 20 | 13,6 | 26,6 | 21,7 | 31,5 | 9,32 | 2,00 |
| 21 | 16,1 | 36,9 | 18 | 29,3 | 22,3 | 1,28 |
| 22 | 8,47 | 14,2 | 23,2 | 47,9 | 2,39 | 5,01 |
| 23 | 15,1 | 32,4 | 23,8 | 31,4 | 6,14 | 1,70 |
| 24 | 12 | 25,7 | 10,6 | 31 | 5,61 | 1,62 |
| 25 | 11,8 | 50,3 | 38 | 8,5 | 5,31 | 0,92 |

Supplementary table 2. Detailed patients’ biological characteristics

*Tfh: frequency of CXCR5+CD45RA- Tfh within CD4+ Tcells; Tfh1: frequency of Tfh1 cells within Tfh; Tfh2: frequency of Tfh2 cells within Tfh; Tfh17: frequency of Tfh17 cells within Tfh; Tfr: frequency of Tfr cells within CXCR5+CD45RA- CD4+ T cells; Ratio: (Tfh2+Tfh17)/Tfh1 ratio.*
