# Supplementary material for: The Balance in T Follicular Helper Cell Subsets Is Altered in Neuromyelitis Optica Spectrum Disorder Patients and Restored by Rituximab
Source: Front Immunol. 2019 Nov 19;10:2686. doi: 10.3389/fimmu.2019.02686 (PMC6877601; doi:10.3389/fimmu.2019.02686)
Supplement: Supplementary file 6 [file Table_3.docx]

Supplementary table 3. Detailed healthy controls’ biological characteristics

| Healthy controls | Tfh | Tfh1 | Tfh2 | Tfh17 | Tfr | Ratio |
| --- | --- | --- | --- | --- | --- | --- |
| 1 | 11.7 | 27.5 | 24.1 | 35.7 | 7.48 | 2.17 |
| 2 | 7.63 | 38.5 | 24.1 | 23.8 | 12.9 | 1.24 |
| 3 | 8.71 | 28.2 | 19.9 | 35.3 | 7.49 | 1.96 |
| 4 | 9.77 | 25.4 | 23.5 | 33.8 | 7.65 | 2.26 |
| 5 | 13.6 | 22.1 | 30.3 | 36.5 | 4.52 | 3.02 |
| 6 | 8.82 | 36.4 | 20.7 | 24.6 | 7.28 | 1.24 |
| 7 | 5.85 | 38 | 18 | 28.8 | 10.7 | 1.23 |
| 8 | 11.8 | 34.3 | 16.8 | 30.5 | 6.38 | 1.38 |
| 9 | 12,5 | 35,3 | 24,8 | 26,2 | 9,4 | 1,44 |
| 10 | 16.3 | 26.2 | 42.8 | 25.9 | 10.1 | 2.62 |
| 11 | 9.01 | 32.9 | 26.8 | 27 | 4.23 | 1.63 |
| 12 | 11.4 | 38.1 | 43.1 | 14.8 | 7.41 | 1.52 |
