# Supplementary material for: The Balance in T Follicular Helper Cell Subsets Is Altered in Neuromyelitis Optica Spectrum Disorder Patients and Restored by Rituximab
Source: Front Immunol. 2019 Nov 19;10:2686. doi: 10.3389/fimmu.2019.02686 (PMC6877601; doi:10.3389/fimmu.2019.02686)
Supplement: Supplementary file 7 [file Table_4.docx]

Supplementary table 4. Antibody reference

| Antibody | Fluorochrome | Supplier | Clone | Dilution |
| --- | --- | --- | --- | --- |
| Anti-human CD3 | FITC | eBioscience, San Diego, CA, USA | OKT3 | 1:5 |
| Anti-human CD4 | PerCpCy5.5 | BD Pharmingen, San Diego, CA, USA | RPA-T4 | 1:5 |
| Anti-human CD45RA | APC-H7 | BD Pharmingen, San Diego, CA, USA | HI100 | 1:5 |
| Anti-human CXCR5 | BV510 | BD Pharmingen, San Diego, CA, USA | RF8B2 | 1:5 |
| Anti-human CD25 | PE | Biolegend, ImTec Diagnostics N.V., Antwerp, Belgium | M-A251 | 1:5 |
| Anti-human CD127 | A647 | Biolegend, ImTec Diagnostics N.V., Antwerp, Belgium | A019D5 | 1:5 |
| Anti-human CCR6 | PeCy7 | Biolegend, ImTec Diagnostics N.V., Antwerp, Belgium | G034E3 | 1:5 |
| Anti-human CXCR3 | BV421 | Biolegend, ImTec Diagnostics N.V., Antwerp, Belgium | G025H7 | 1:5 |
