# Supplementary material for: The Balance in T Follicular Helper Cell Subsets Is Altered in Neuromyelitis Optica Spectrum Disorder Patients and Restored by Rituximab
Source: Front Immunol. 2019 Nov 19;10:2686. doi: 10.3389/fimmu.2019.02686 (PMC6877601; doi:10.3389/fimmu.2019.02686)
Supplement: Supplementary file 8 [file Table_5.docx]

Supplementary table 5 : Compensation matrix

|  | CD3-FITC | CD25-PE | CD4-PerCpCy5.5 | CCR6-PECy7 | CD127-A647 | CD45RA-APC-H7 | CXCR3-BV421 | CXCR5-BV510 |
| --- | --- | --- | --- | --- | --- | --- | --- | --- |
| CD3-FITC | 100 | 13.9 | 1.5 | 0 | 0 | 0 | 0 | 1.5 |
| CD25-PE | 0 | 100 | 15.9 | 1 | 0 | 0 | 0 | 0 |
| CD4-PerCpCy5.5 | 0 | 0 | 100 | 24 | 2 | 6.8 | 0 | 0 |
| CCR6-PECy7 | 0 | 0.5 | 2.1 | 100 | 0 | 11 | 0 | 0 |
| CD127-A647 | 1 | 0 | 0 | 0 | 100 | 20 | 0 | 0.8 |
| CD45RA-APC-H7 | 0 | 0 | 0 | 1.2 | 1 | 100 | 0 | 0 |
| CXCR3-BV421 | 0 | 0 | 0 | 0 | 0 | 0 | 100 | 10 |
| CXCR5-BV510 | 0 | 0 | 0 | 0 | 0 | 0 | 10 | 100 |
